# Types of Resin Composite and Filling Techniques: How They Affect Internal Void Volume and Compressive Strength

**DOI:** 10.3390/polym18070885

**Published:** 2026-04-04

**Authors:** Pirat Karntiang, Chitpol Chaimanakarn, Daranee Chaimanakarn, Thanyaporn Niyomdee, Hiroshi Ikeda

**Affiliations:** 1 Division of Operative Dentistry, College of Dental Medicine, Rangsit University, Pathum Thani 12000, Thailand; 2 Endodontic Department, Faculty of Dentistry, Mahidol University, Bangkok 10400, Thailand; 3 Division of Biomaterials, Kyushu Dental University, Fukuoka 803-8580, Japan

**Keywords:** resin composite, bulk-fill resin composite, incremental technique, internal void, compressive strength, micro-CT

## Abstract

Background: This study evaluated the void volume percentage and compressive strength of restorations prepared with different resin composite materials and techniques. Methods: There were five experimental groups: (1) ZS (Filtek™ Z350XT, single layer); (2) ZH (Filtek™ Z350XT, horizontal increment); (3) ZO (Filtek™ Z350XT, oblique increment); (4) BS (Filtek™ One Bulk Fill, single layer); (5) BF (Filtek™ One Bulk Fill and Filtek™ Z350XT Flowable Composite). Specimen dimensions were 4 mm high and 5 mm in diameter. Internal void evaluation was done by micro-CT, followed by a compressive strength test. Data were analyzed using one-way analysis of variance (ANOVA) followed by Tukey’s post hoc test (*p* = 0.05) and Pearson’s correlation analysis. Results: Group BF had the largest void volume percentage (0.5501 ± 0.2031%), and groups ZS and BS had the smallest (0.0366 ± 0.0279% and 0.1991 ± 0.1463%, respectively). Groups ZH and ZS showed significantly higher compressive strength values (200.18 ± 16.32 MPa, 192.18 ± 17.23 MPa, respectively) than groups BS and BF. Pearson’s correlation analysis revealed no statistically significant correlation. Conclusions: The multilayer filling technique created significantly more voids than the single-layer technique. Some materials also contain inherent voids. However, these voids may not directly affect the strength of the restoration.

## 1. Introduction

Resin composites are the preferred material for direct tooth restoration in modern dentistry due to their excellent physical, mechanical, and optical properties [1]. These materials have been extensively researched and developed over the past decades, both mechanically, chemically, and aesthetically [2]. The modern paste-type resin composite, which can be used for restorations in both anterior and posterior teeth, is generally known as a universal resin composite [3]. Numerous studies have been published on filling techniques aimed at minimizing potential problems caused by restorative procedures, primarily to improve the overall properties of the restorations [4,5,6,7,8]. The main limitation of this type of resin composite relates to polymerization shrinkage and depth of cure. These two factors require the dentist to place the material into the cavity in increments of 2 mm per layer. Currently, two primary techniques are extensively employed: the horizontal incremental technique and the oblique incremental technique. These two techniques modified the cavity configuration, thereby reducing the C-factor [9,10] and became the preferred method for composite applications in most cavities [11,12]. Nevertheless, this technique appeared to be associated with an increased risk of void formation at interfaces between layers [13,14], especially when restoring large, deep, and difficult-to-access cavities [12,15].

Voids in restorations can weaken resin composites by trapping air, reducing their flexural strength and fatigue resistance, and increasing susceptibility to wear under occlusal forces [16,17,18]. Furthermore, internal voids may reduce the durability of the composite, potentially leading to fractures and adverse clinical outcomes in restorative procedures [19].

A so-called bulk-fill resin composite was released to the market to compete with the time-consuming incremental technique [20,21]. The manufacturer claims that this composite can be light-cured to a 4–5 mm layer [3,22], thereby saving about half the time and reducing interlayer void formation. Reports have indicated that bulk-fill resin composites reduce the incorporation of voids, potentially attributable to diminished handling requirements during the restorative process and fewer interfaces [7,12,13,14,23]. Nonetheless, they demonstrated outcomes comparable to conventional ones [24]. However, there were studies reporting higher porosity in thicker material [25] and interfacial gap formation between the tooth structure and the resin composite [26,27,28]. Hence, some publications recommended using a bulk-fill resin composite with a flowable resin composite liner [29,30].

Although bulk-fill resin composite appears to overcome the limitations of filling large and deep cavities, there remains inconclusive evidence regarding the percentage of void volume in bulk-fill resin composite restorations compared with universal resin composite restorations performed with the incremental technique. In addition, the relationship between void volume and compressive strength has not yet been confirmed.

The materials investigated in this study included resin composites commonly indicated for both anterior esthetic restorations [31] and posterior load-bearing applications [32]. Therefore, comparing these materials enables the assessment of how variations in composition and handling properties may affect void formation and mechanical performance under different clinical indications.

The aim of this research was to: (1) evaluate the void volume percentage, (2) evaluate the compressive strength of resin composite prepared with different combinations of materials and filling techniques, and (3) determine whether there is a relationship between the void volume and compressive strength.

## 2. Materials and Methods

### 2.1. Specimen Preparation

The required sample size was calculated using G*Power software version 3.1.9.7. The calculation relied on parameters derived from a study by Gigova R. and Hristov K. [21]. For an ANOVA, with a significance level of 0.05 and a power of 0.95, the analysis indicated that a total sample size of 50 (*n* = 10 per group) was sufficient. The actual power achieved for this setup was 0.9565.

The materials used in the study, including their chemical compositions, batches, and manufacturers, as well as the devices used for specimen fabrication or evaluation, are listed in Table 1. There was one control group and four experimental groups: (1) ZS (Filtek Z350XT in 4 mm single layer—control), (2) ZH (Filtek Z350XT in 2 horizontal layers), (3) ZO (Filtek Z350XT in 4 oblique layers), (4) BS (Filtek One Bulk Fill in 4 mm single layer), and (5) BF (Filtek One Bulk Fill lined with 1 mm layer of flowable composite). Specimens with a 5 mm diameter and a 4 mm height were fabricated using a stainless-steel split mold.

The resin composite types and the detailed procedures for filling techniques are described in Table 2. Following the leveling of the specimen surface, a mylar strip was positioned onto the surface of the specimen. Each light activation was carried out using Demi™ Plus (Kerr, USA), which was positioned perpendicular to the specimen surface at the top of the mold, in contact with the mylar strip, for a duration of 40 s. All restorative procedures were performed by one operator at a constant room temperature (25 °C). The specimens were subjected to micro-CT scanning and stored in distilled water at 37 °C for 24 h prior to the compressive strength test.

### 2.2. Micro-CT Scanning and Void Analysis

Two examiners performed the scanning and interpretation process in a blinded manner. The specimens were analyzed using Skyscan 1173 computed X-ray microtomograph (Bruker, Kontich, Belgium). The scanning parameters were set to a voltage of 80 kV, current of 100 μA, a 1.0 mm aluminum filter, a pixel size of 9.86 μm, and a rotation step of 0.3°, a full specimen rotation of 360°, and an exposure time of 950 ms for each projection. The average duration for each scan was approximately 40 min. Other settings, including beam-hardening correction and optimal contrast input, were in accordance with the instructions.

The NRecon (version 1.7.5.1, Bruker, Billerica, MA, USA) and CTAn (CT-Analyser version 1.18.8.0, Bruker, Billerica, MA, USA) were used to convert the specimen projection into axial slices with a resolution of 2016 × 2016 pixels. The reconstruction parameters were as follows: ring artifact correction and misalignment were set to zero, and beam hardening correction was set to 39% by finding the best frames. The images obtained from the scanner were reconstructed to show 2D slices of the restoration, totaling 1531 cross-sectional images from the whole volume.

Prior to data analysis, DataViewer (SkyScan, version 1.5.6.5) was used to adjust the alignment of each specimen from multiplanar reconstruction. Then, CTAn software was used to visualize, analyze, and measure the 3D volumes of the restorations. In each section, 2D slices were used to evaluate the presence of voids. Two examiners simultaneously evaluated each segment using a binary scale to assess internal voids in micro-CT images. Each section was re-examined by the examiners until agreement was reached.

A thresholding (binarization) process was used to convert the range of gray levels into an image containing only black and white pixels. For each specimen, a region of interest (ROI) was defined using consistent protocols to ensure that each slice captured within the boundary of the specimen, enabling the calculation of void volumes. The ROI was circular, with a diameter of 4.5 mm and a height of 3.30 mm. The number of images within the VOI was 336. All specimens ultimately had the same final volume of 52.42 mm^3^. Then, the percentage of internal void volume within each specimen was calculated.

### 2.3. Compressive Strength Evaluation

After micro-CT analysis, all specimens were subjected to a compressive strength test using a universal testing machine (Instron model 5566, Canton, MA, USA) at a crosshead speed of 1 mm/min. A flat circular crosshead and a 1 kN load cell were used. A piece of tin foil was placed between the crosshead tip and the specimens to help distribute the force if the surfaces were not parallel and to prevent chipping of the specimen edges. The crosshead of the testing machine stopped when a sudden drop occurred on the recording chart due to catastrophic failure. All compressive strengths were recorded in megapascals (MPa).

### 2.4. Statistical Methods and Data Processing

The Kolmogorov–Smirnov and Levene’s test were used to assess the normality and variance homogeneity. One-way ANOVA followed by Tukey’s post hoc test was conducted to detect significant differences among groups and for pairwise comparisons. A significance level of 5% (*p* = 0.05) was applied. The relationship between void volume percentage and compressive strength was assessed using Pearson’s correlation coefficient (*p* = 0.05). All statistical analyses were performed with EZR version 1.70 (Aichi Medical University, Aichi, Japan).

## 3. Results

**Figure 1:**
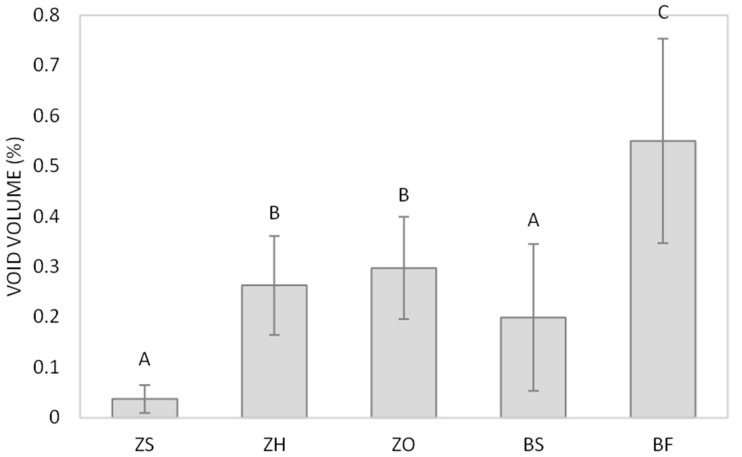
Void volume percentage in resin composite. Different uppercase letters indicate significant differences between groups (*p* < 0.05, Tukey’s test, *n* = 10).

**Figure 2:**
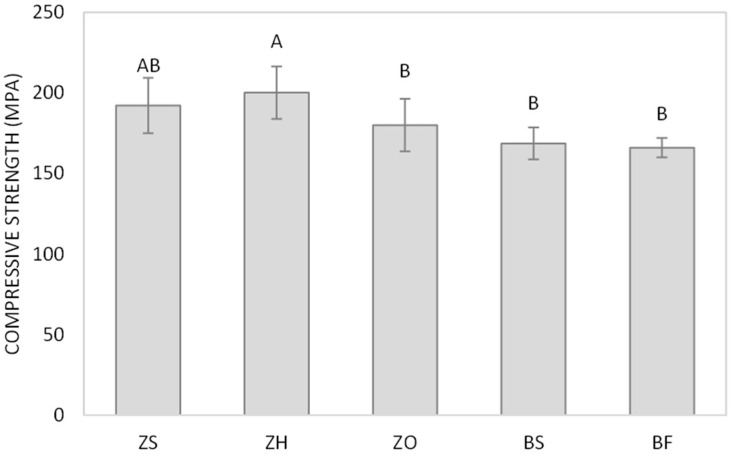
Compressive strength of resin composite. Different uppercase letters indicate significant differences between groups (*p* < 0.05, Tukey’s test, *n* = 10).

Table 3, Figure 1 and Figure 2 show the mean void volume (%) and mean compressive strength (MPa). Group BF had the highest void volume (0.5501 ± 0.2031%). Group ZS had the lowest void volume (0.0366 ± 0.0279%), followed by group BS (0.1991 ± 0.1463%). Though there was no statistical difference between groups ZS and BS (*p* > 0.05). Group ZH and ZO showed higher void volume than group BS (0.263 ± 0.0984% and 0.2974 ± 0.1018%, respectively). Also, there was no statistical difference among groups BS, ZH, and ZO.

**Figure 3:**
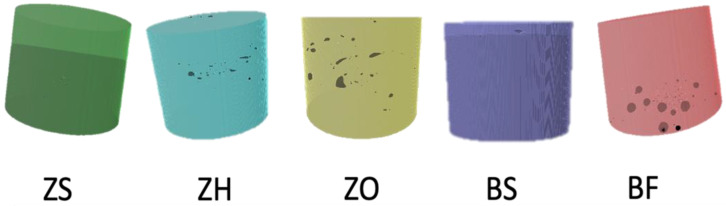
3D rendering of one representative specimen of each composite showing the presence of internal void.

Three-dimensional renderings of representative samples from each group were reconstructed (Figure 3). The internal void was predominantly observed in the middle region of group ZH, whereas in group ZO, the void was distributed both horizontally and obliquely along the interface. Voids in these two groups exhibited heterogeneity in shape. Group BF, conversely, exhibited a spherical void in the bottom part of a flowable resin composite. Groups ZS and BS exhibited minimal presence of voids in the renderings.

For compressive strength, groups ZH and ZS showed the highest values (200.18 ± 16.32 and 192.18 ± 17.23 MPa, respectively), with no significant difference between groups. Whereas groups ZO (180.01 ± 16.21 MPa), BS (168.59 ± 9.92 MPa), and BF (166.04 ± 5.97 MPa) showed no statistically significant differences among groups.

A Pearson’s correlation analysis revealed no statistically significant relationship (*r* = −0.073) between void volume percentage and the compressive strength (*p* > 0.05).

## 4. Discussion

Regarding specimen configuration and dimension, the specimen was cylindrical to match the micro-CT scanning area and facilitate fabrication. Although a rounded-corner-rectangle column would be more representative of a clinical situation, it was not suitable for the compressive strength test. The mold was 4 mm high with a 5 mm diameter, resembling a moderately deep cavity. In practice, a layer of resin composite no thicker than 2 mm should be placed incrementally to ensure optimal polymerization and degree of conversion [33].

Group ZS, which is not applicable in real clinical settings, served as the control group and was deliberately devoid of an interface. However, due to the limited curing depth of the material, this group required light activation from both the top and bottom for 40 s on each side. Finally, group ZS received the same amount of light activation as that of group ZH, which was considered sufficient for optimal polymerization.

To mitigate inconsistencies arising from the restorative skill of multiple operators, the specimens were fabricated by a trained single operator utilizing a stainless-steel mold. This approach decreased the restorative workload and standardized variables such as cavity size, shape, and depth to minimize human error. The mold also helped ensure that the light-curing unit was positioned at a consistent height of 5 mm every time.

The light-curing device employed in this research (Demi™ Plus) was a periodic level-shifting variant, characterized by alternating light intensities of 1100 mW/mm^2^ and 1330 mW/mm^2^. The device was recharged after each curing cycle to ensure optimal output. To ensure a curing depth of 4 mm for the more viscous bulk-fill resin composite, a light activation time of 40 s was also used for the bulk-fill resin composite [22].

This study utilized micro-computed tomography (micro-CT) to evaluate the internal void volume, as micro-CT was recognized for its ability to provide exceptional detail, facilitating analysis through computer software [6,21,28,34,35]. During the analysis, two independent analysts examined the samples to ensure accuracy and objectivity. In a binary registration process, they could adjust the threshold until consensus was reached. Moreover, micro-CT was a non-destructive analytical technique, allowing for subsequent examination of the specimens.

Multiple studies have demonstrated that incremental techniques contribute to void formation, as air bubbles tend to accumulate in the space between the overlying increment and the polymerized increments [7,34,36,37]. To ascertain whether different filling techniques influence void formation, both horizontal and oblique incremental techniques were examined.

Group ZO, using the oblique incremental technique, with the most interfaces (4 layers, 3 interfaces), had the highest void volume percentage within the same material groups. Voids were predominantly at interfaces, as shown by 3D reconstruction (Figure 3). In addition, sculpting and manipulating each layer were unavoidable during layer filling. Thus, there is an increased likelihood of air entrapment. The results of the current study showed that the restoration with more interfaces exhibited a higher void volume percentage. The findings were consistent with those of a previous study [7,34,36,37]. Demirel G concluded that every new increment was a source of trapped air [7]. However, there was no significant difference between the ZH and ZO groups.

The data from the current study showed that the single-layer application groups, ZS and BS, both exhibited significantly lower void volume percentages than the multiple-layer groups. Applying a high-viscosity resin composite as a single layer can help prevent voids, as no interface forms and less handling occurs during application. As a result, air trapping was minimized [34]. However, group BS exhibited a slightly higher void percentage, with a large SD. This might be due to the operator’s unfamiliarity with the viscosity of the material and the inherent voids from the manufacturing process [28,38]. As a result, the void volume of group BS increased with a large SD even though this group had no interface.

Comparing groups BS and BF, BS had a significantly lower void volume percentage than BF. Although group BF had only one interface, it showed the highest void volume percentage. The 3D reconstruction revealed that the voids were mostly in the flowable resin composite layer, and then at the interface. Díaz C et al. also reported that the use of a flowable resin composite increased the void volume percentage [8]. This could be explained by the chemical components of the low-viscosity flowable resin composite. By adding a significant amount of low-molecular-weight monomers without an aromatic core or accessible hydroxyl groups, low-viscosity resin composites can be created. The most common monomer is triethylene glycol dimethacrylate (TEGDMA), which serves as a diluent. The viscosity of the material also decreases as the filler volume fraction decreases. However, porosity exists in the matrix, the continuous phase, rather than in the dispersed phase. As a result, a material with less filler may theoretically be more porous on a proportional basis. It is known that the polymer network formed by TEGDMA is more heterogeneous than that formed by Bis-GMA. Between polymer clusters, this heterogeneity tends to enhance the occurrence of microporosities as well [39]. Increased void formation following the injection of low-viscosity resin materials has been documented in several studies [8,34]. Therefore, it should be noted that low-viscosity materials may contain inherent voids that contribute to the overall void volume in the restoration.

Regarding compressive strength, this study reported data that were comparable yet significantly different. Group ZH exhibited significantly higher compressive strength than groups ZO, BS, and BF, but not significantly different from group ZS. The major factors contributing to the compressive strength of the resin composite were the resin matrix and the filler [40,41,42]. The type of monomer, degree of conversion, and crosslinking all contributed to the strength of the material [43]. In addition, the type, size, and amount of incorporated filler, as well as the quality of the filler surface silanization, were important for strengthening the resin composite [41,43,44,45,46,47]. The significant difference in compressive strength between groups ZH and ZO might be due to the higher void volume resulting from doubling the number of resin composite layers in group ZO, which increased the void volume percentage.

In the current study, the percentage of void volume appeared not to influence compressive strength, as Pearson’s correlation analysis revealed no statistically significant relationship. The findings suggest that variations in void content did not markedly affect the mechanical strength of dental restorations. This may be attributed to the small size and quantity of the voids, as well as the inherent strength of the resin composite utilized in this investigation. Nevertheless, a noticeable decline in the average compressive strength was observed as the filling technique shifted from a single-layer to a four-layer approach. This numerical trend consistently aligns with the increase in void content documented in this study.

Nevertheless, internal voids have the potential to compromise the mechanical integrity of composite materials, thereby diminishing their strength and increasing the likelihood of fracture. Chadwick R. et al. reported a reduction in the compressive strength of resin composite when produced using a technique that resulted in a higher number of internal voids [48]. McCabe J. and Ogden A. also concluded that internal voids may significantly influence fatigue resistance and impact durability [49]. Furthermore, over a period, these voids may serve as points of stress concentration, induce crack formation, and potentially lead to the failure of the restoration [50].

However, this in vitro study had several limitations that need to be addressed. First, the mold-made restoration lacked the supporting surrounding tooth structure and was unable to accurately replicate the shape of the prepared cavity. The decision to exclude the extracted tooth from this research was made to eliminate potential confounding factors that could obscure the results and correlations. Second, environmental conditions, including temperature, humidity, and the presence of dentinal fluid, were not consistent with those in the oral cavity. Such factors can influence the formation of internal voids, thereby constraining the clinical relevance of the findings. Third, the study did not incorporate aging simulations such as thermocycling, which could potentially exacerbate the effects of internal voids and thereby accentuate the differences. These unexamined variables may also impact material performance and void formation. Fourth, although two trained micro-CT analysts assessed internal voids without knowing the specimen groups, they might have inadvertently recognized them because the void distribution was apparent in some groups.

Therefore, it is recommended to conduct further research using extracted human teeth to examine additional relevant mechanical properties in relation to the degree of conversion, and to perform artificial-aging simulations or fatigue loading, followed by load-to-failure, to confirm the applicability of the findings in clinical practice.

## 5. Conclusions

Within the limitations of this in vitro study, it can be concluded that both the filling technique and material characteristics affect internal void volume percentages. The incremental technique has a higher likelihood of creating voids than the single-layer method. Additionally, combining flowable resin composite with bulk-fill resin composite increases the total void count. Although there was no significant correlation between increased void volume percentage and decreased compressive strength in this study, it is still crucial to perform restorations with as few voids as possible to ensure optimal mechanical properties and longevity.

Clinical Implications: Concerning the incremental technique utilizing universal resin composite, the primary cause of internal voids is the interface between successive layers. Bulk-fill resin composite presents a feasible alternative to mitigate void formation at the interface. Employing a flowable resin composite to enhance adaptation to the cavity wall is recommended; however, it is advisable to limit its use, owing to inherent voids resulting from the manufacturing process.

## Figures and Tables

**Table 1:** Materials and devices.

Materials/Devices	Compositions/Descriptions	Lot No.
Filtek™ One Bulk Fill Restorative	AFM (dynamic stress-relieving monomer), AUDMA, UDMA, and 1,12-dodecane-DMA Fillers: a combination of a non-agglomerated/non-aggregated 20 nm silica filler, a non-agglomerated/non-aggregated 4 to 11 nm zirconia filler, an aggregated zirconia/silica cluster filler (comprising 20 nm silica and 4 to 11 nm zirconia particles), and a ytterbium trifluoride filler consisting of agglomerate 100 nm particles. The inorganic filler loading is about 76.5% by weight (58.5% by volume). (3 M Oral Care, St Paul, MN, USA)	9189369
Filtek™ Z350XT Flow	Bis-GMA, TEGDMA, and bis-EMA Fillers: a combination of 5 nm diameter of non-agglomerated/non-aggregated silica nanofiller, 5–10 nm diameter of non-agglomerated/non-aggregated zirconia nanofiller, loosely bound agglomerated zirconia/silica nanocluster, consisting of agglomerates of 5 to 20 nm primary zirconia/silica particles and a cluster particle size range of 0.6 to 1.4 microns. The inorganic filler loading is about 65% by weight (55% by volume). (3 M Oral Care, St Paul, MN, USA)	NF42375
Filtek™ Z350XT	Bis-GMA, Bis-EMA, UDMA and TEGDMA Filler: 59.5 vol.% combination of aggregated zirconia/silica cluster ranging from 0.6 to 1.4 µm with primary particle size of 5–20 nm and non-agglomerated 20 nm silica filler. (3 M Oral Care, St Paul, MN, USA)	NF31573
Demi™ Plus	An LED light curing device. (Kerr, Orange, CA, USA)	
Bruker Skyscan 1173	A desktop Micro-CT system (Kontich, Belgium). X-ray source is 40–130 kV, 8 W. The X-ray detector is a distortion-free flat panel sensor (2240 × 2240). The resolution is 5 microns.	
Instron model 5566	A universal testing machine (Canton, MA, USA)	

Abbreviations: Bis-GMA, bisphenol A diglycidyl methacrylate; UDMA, urethane dimethacrylate; TEGDMA, triethylene glycol dimethacrylate; bis-EMA, bisphenol A ethoxylated dimethacrylate; AFM, addition-fragmentation chain transfer monomer; AUDMA, aromatic urethane dimethacrylate; UDMA, urethane dimethacrylate; DMA, dimethylacetamide.

**Table 2:** Experimental groups, materials used, and the methods of filling technique.

Group	Material and Filling Technique	Application Method and Illustration	Number of Layers
ZS	Filtek Z350 Single layer	A 4 mm resin composite layer was placed in a mold on a glass slab. The surface was leveled with an IPC instrument and light-cured from top and bottom for 40 s each. 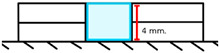	1
ZH	Filtek Z350 Horizontal increment	A 2 mm resin composite layer was placed in a mold on a glass slab. The surface was leveled with an IPC instrument and light-cured for 40 s from the top. This was repeated to create a 4 mm specimen. 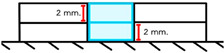	2
ZO	Filtek Z350 Oblique increment	A 2 mm resin composite layer was placed in a mold on a glass slab. Its top was leveled diagonally with an IPC instrument and light-cured for 40 s. Another layer was applied, leveled horizontally with an IPC hand instrument, and light-cured for 40 s. This process was repeated to create a 4 mm high resin specimen. 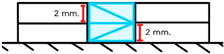	4
BS	Filtek One Bulk Fill Single layer	A 4 mm layer of bulk-fill resin composite was placed in a mold on a glass slab. The top was leveled with an IPC instrument and light-cured for 40 s. 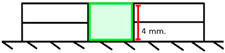	1
BF	Filtek One Bulk Fill with flowable resin composite lining	A 1 mm layer of flowable resin composite was placed in a mold and light-cured for 40 s. Then, a 3 mm layer of bulk-fill resin was applied, leveled with an IPC instrument, and light-cured for 40 s. 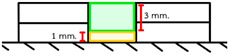	2

**Table 3:** Mean percentage of void volume and compressive strength.

Group	Void Volume (%)	Compressive Strength (MPa)
ZS	0.0366 ± 0.0279 ^A^	192.18 ± 17.23 ^ab^
ZH	0.263 ± 0.0984 ^B^	200.18 ± 16.32 ^a^
ZO	0.2974 ± 0.1018 ^B^	180.01 ± 16.21 ^b^
BS	0.1991 ± 0.1463 ^A^	168.59 ± 9.92 ^b^
BF	0.5501 ± 0.2031 ^C^	166.04 ± 5.97 ^b^

Different uppercase and lowercase letters indicate significance between groups.

## Data Availability

The original contributions presented in this study are included in the article. Further inquiries can be directed to the corresponding author.
